# Graft survival differences in kidney transplants related to recipient sex and age

**DOI:** 10.3389/fmed.2022.962094

**Published:** 2022-09-26

**Authors:** Asuncion Sancho, Eva Gavela, Julia Kanter, Sandra Beltrán, Cristina Castro, Verónica Escudero, Jonay Pantoja, Pablo Molina, Belen Vizcaíno, Mercedes González, Emma Calatayud, Ana Avila

**Affiliations:** ^1^Department of Nephrology, Hospital Universitari Dr Peset, Valencia, Spain; ^2^Department of Medicine, University of Valencia, Valencia, Spain; ^3^Fundación para el Fomento de la Investigación Sanitaria y Biomédica de la Comunitat Valenciana (Fisabio), Valencia, Spain

**Keywords:** gender disparities, kidney transplantation, female recipients, graft survival, patient survival

## Abstract

**Background:**

In recent years, there has been increasing interest in studying differences in recipient sex in renal disease treatment, access to renal replacement therapy, and subsequent outcomes. Our aim was to find out whether there are differences in outcomes after renal transplantation between female and male kidney transplant recipients in our series, particularly in adults under 60 years of age during long-term follow-up.

**Methods:**

This was a retrospective study of our kidney transplant series (*n* = 1,101) to compare graft survival depending on the sex of the recipient in the entire series and patients < 60 years of age (*n* = 687) during long-term follow-up.

**Results:**

We observed no association between recipient sex and graft survival throughout the series, regardless of recipient sex. However, adult female recipients under 60 years of age had lower graft survival than male recipients (*p* = 0.040). Pre-transplant sensitization (HR 2.438, *p* = 0.002) and donor age (HR: 1.021, *p* = 0.017) were the independent variables associated with graft failure.

**Conclusion:**

Female recipients younger than 60 years of age had lower graft survival than male recipients, although there were no gender differences in graft or patient survival in the overall study population. Recipient sex *per se* was not related to graft failure, but the greater immunological risk in women and more frequent use of expanded criteria donors in female recipients under 60 years of age were the main factors related to their poorer graft survival. Further studies and new strategies are needed to identify these differences and develop the best approach to address them.

## Introduction

There is growing evidence that there are inequalities in various aspects of kidney disease, such as the burden of kidney disease, access to renal replacement therapies (RRT), or their subsequent development, in relation to the gender of the recipient (1–3). A higher burden of chronic kidney disease (CKD) and a higher proportion of pre-dialysis CKD have been described in women compared to men, but fewer women start renal replacement therapy, and female adult patients, as well as children, have been described as having poorer access to deceased and living donor transplants (1–3). Various biological, psychological, and socioeconomic aspects have contributed to gender differences in kidney disease recipients, but their detection appears to be more difficult than racial or economic aspects (4, 5).

Several authors and large registries have found no difference in survival between men and women after kidney transplantation, although the effect of the recipient’s sex on graft survival has been disputed in several studies (1, 4, 6). The aim of this study was to find out whether there are differences in long-term graft survival and associated risk factors in female recipients, particularly in adult recipients younger than 60 years of age, for whom achieving a longer graft survival rate is critical to avoid the need for a new graft in the event of graft failure.

## Materials and methods

### Data source and study population

We performed a retrospective analysis on 1,101 adult deceased and living donor kidney transplant recipients at our hospital from January 2000 to January 2019. None of them was a multi-organ transplant. The allocation process during the period of analysis was not based on a computer algorithm but the independent decision of the nephrology staff. Patients gave signed consent for the use of their clinical and personal data for educational and research purposes. Data were taken from our kidney transplant database, which includes variables on donor and recipient demographics, kidney function, infections, cardiovascular disease, development of cancer, and graft failure or death and includes all patients transplanted since the start of our kidney transplant program. All data were extracted from each patient’s medical records and entered into the database by trained personnel. The data were kept confidential in accordance with Spanish law and the Declaration of Helsinki.

### Variables analyzed

We analyzed the demographic characteristics of the recipients, such as sex, age, dialysis modality [preemptive transplantation, hemodialysis (HD), peritoneal dialysis (PD)], dialysis time (in months), re-transplantation, previous HLA sensitization, pre-transplant comorbidities such as hypertension (HTN), diabetes mellitus (DM), smoking habits or ischemic cardiomyopathy, body mass index (BMI), and end-stage renal disease (ESRD) etiology. Donor demographics such as sex, age, type of donor brain death donor (DBD), donor after circulatory death [DCD type II or type III, pediatric block or living donor (LD)], cerebrovascular death, HTN, and serum creatinine were analyzed. Duration of cold ischemia (hours), immunosuppressive treatment (tacrolimus), delayed graft function (DGF), acute rejection (AR) during the first 6 months diagnosed by biopsy, etiology of graft failure, and patient death were also studied.

Initially, we considered HLA sensitization in patients with PRA higher than 10% and, after 2012, in patients with anti-HLA higher than 1,500 MFI (Luminex techniques).

The immunosuppressive regimen included therapy with a standard dose of a calcineurin inhibitor (tacrolimus or cyclosporine), m-Tor inhibitors (sirolimus or everolimus), mofetyl mycophenolate, or sodium mycophenolate, and steroids in a decreasing dosage. Induction treatment with interleukin-2 receptor antagonist was used in non-extended criteria donors and low immunological risk patients. Thymoglobulin in reduced doses (two to three doses of 1.25 mg/kg every other day) was used for induction in the case of extended criteria donors with a high risk of DGF and a maximum cumulative dose of 6 mg/kg in those patients with high immunological risk (7).

### Statistical analysis

Initially, a descriptive analysis of donor and recipient demographics and post-transplant variables was performed. Qualitative variables were described with absolute frequencies and percentages. Quantitative variables were summarized as the mean and standard deviation. A bivariate analysis to compare donor and recipient characteristics as well as the post-transplant variables, depending on the recipient sex, was performed. Associations between qualitative variables were evaluated by Fisher’s exact test. Based on the recipient sex, the Student’s *t*-test was used to compare the quantitative variables that were normally distributed, and the Mann-Whitney test was used for the non-normally distributed ones. Normality was tested with the Shapiro-Wilk test and the Bartlett test. Graft survival according to recipient sex was calculated using the Kaplan-Meier method, and the null hypothesis was tested using the Log-rank test.

Additionally, Cox regression models were used to evaluate the time to graft failure depending on recipient and donor characteristics. The following were considered as possible explanatory variables in the model: sex, age, BMI, pre-transplant HLA-sensitization, donor sex and age, cardiovascular disease as the cause of donor death, cold ischemia time, and AR episodes in the first 6 months after transplantation. Goodness-of-fit of the models was evaluated by calculating their concordance, that is, the degree to which the models distinguish between patients at higher risk and lower risk (1 would indicate perfect discrimination; 0.5 would indicate discrimination close to chance). The score test was used to verify the proportional risk assumption (8). All the analyses were performed on the whole study population and younger recipients (under 60 years of age). We stratified our study population at 60 years of age, following other studies that consider differences in immune response and kidney transplant evolution at this age (9, 10).

R software version 4.0.5 (R Foundation for Statistical Computing, Vienna, Austria) was used to perform statistical analysis. *P*-values less than 0.05 (two-tailed) were considered statistically significant.

## Results

### Female recipients in the whole study population

The whole study population included 1,101 kidney transplant recipients with a mean follow-up of 7.2 ± 5.9 years (interquartile range: 2 – 11.1), of whom 435 (39.5%) were female recipients and 666 (60.5%) were male recipients.

**FIGURE 1:**
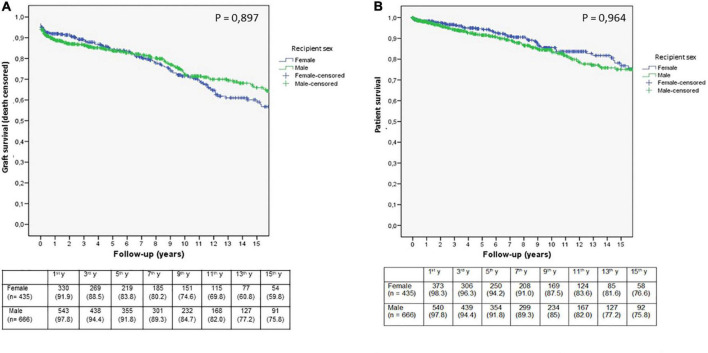
Kaplan-Meier estimates of death-censored graft **(A)** and patient survival **(B)** according to recipient sex in the whole study population. The number of patients at risk during follow-up is indicated in the table below the figures (*n* = 1,101).

With respect to ESRD origin, interstitial and polycystic kidney diseases were more prevalent in female recipients, while glomerular and vascular diseases were more prevalent in male recipients in the bivariate analysis (Table 1). Tobacco use was less frequent in female recipients. Pre-transplant sensitization was higher in women as well as the use of female donors and DBD. Induction treatment was less frequent in female recipients. No relevant differences were found in the rest of the analyzed variables. Kaplan-Meier curves showed no differences in graft or patient survival depending on recipient sex (Figure 1). The main cause of graft failure was chronic rejection in women and death with functioning graft in men (*p* = 0.014). The main causes of death were cardiovascular diseases and cancer (both of them more frequent in male recipients) and infectious diseases, with no statistical differences (Table 1). The incidence rate of graft failure and death was similar for female recipients (4.9%/year and 1.6%/year, respectively) and male recipients (4.8%/year and 1.9%/year, respectively). In Cox regression, the risk for graft failure increased by 2.7 times in sensitized patients, and the other factors related to graft failure were the occurrence of AR episodes, BMI, and donor age (Table 2). Recipient sex *per se* was not related to graft failure.

**TABLE 1:** Demographic characteristics of recipients, donors, and post-transplant variables depending on recipient sex in the whole study population and the adult recipients younger than 60 years.

	Global series (*n* = 1.101)			Patients younger than 60 years (*n* = 685)		
		
	Female (*n* = 435, 39.5%)	Male (*n* = 666, 60.5%)	*P*	Female (*n* = 277, 40.4%)	Male (*n* = 408, 59.6%)	*P*
**Recipient demographics**						
Age (years) (x ± DS)	53.2 ± 12.3	52.9 ± 13.2	0.81	46.3 ± 9.7	44.8 ± 10	0.051
ESRD etiology (%): Glomerular Interstitial Vascular Polycystic Diabetic nephropathy Systemic Unknown Others	86 (21.1) 79 (19.4) 36 (8.8) 79 (19.4) 12 (2.9) 22 (5.4) 85 (20.9) 8 (2)	176 (28.3) 59 (9.5) 108 (17.4) 83 (13.3) 36 (5.8) 24 (3.9) 122 (19.6) 14 (2.3)	**<0.001**	65 (24.6) 48 (18.2) 20 (7.6) 54 (20.5) 5 (1.9) 20 (7.6) 47 (17.8) 5 (1.9)	131 (33.8) 41 (10.6) 50 (12.9) 58 (14.9) 21 (5.4) 20 (5.2) 56 (14.4) 11 (2.8)	**<0.001**
Dialysis modality (%) Preemptive transplantation Hemodialysis Peritoneal dialysis	2 (0.5) 323 (78.8) 85 (20.7)	14 (2.2) 506 (79.7) 115 (18.1)	0.036	1 (0.4) 198 (74.7) 66 (24.9)	5 (1.3) 315 (79.9) 74 (18.8)	0.41
Months on dialysis (x ± DS)	44.9 ± 43.5	55.5 ± 232.3	0.29	44.9 ± 43.55	45.6 ± 43.4	0.60
HTN pre-Tx (%)	335 (85.5)	516 (87.6)	0.34	219 (84.9)	313 (83.7)	0.74
DM pre-Tx (%)	32 (8.3)	64 (10.0)	0.19	17 (6.6)	25 (6.7)	0.97
Tobacco use pre-Tx (%)	113 (30.3)	315 (55.5)	**<0.001**	88 (35.9)	195 (54)	**<0.001**
Ischemic cardiomyopathy pre-Tx (%)	19 (5.1)	49 (8.6)	0.053	7 (2.8)	23 (6.3)	0.057
BMI (x ± DS)	25.6 ± 5.2	25.4 ± 3.6	0.74	24.9 ± 5.6	24.9 ± 3.8	0.32
HLA sensitization pre-Tx (%)	78 (19.0)	27 (4.3)	**<0.001**	60 (22.6)	23 (5.9)	**<0.001**
**Donor demographics**						
Female (%)	220 (52)	268 (41.1)	**<0.001**	134 (49.6)	158 (39.4)	**0.011**
Age (years) (x ± DS)	53 ± 19.2	52.6 ± 19	0.56	46.5 (18.1)	44.3 (17.6)	**0.033**
Donor type (%): DBD DCD type II DCD type III Pediatric block LD	380 (87.4) 8 (1.8) 15 (3.4) 22 (5.1) 10 (2.3)	592 (88.9) 16 (2.4) 24 (3.8) 20 (3.0) 13 (2.0)	0.45	235 (84.8) 6 (2.2) 10 (3.6) 17 (6.1) 9 (3.2)	349 (85.5) 16 (3.9) 13 (3.2) 18 (4.4) 12 (2.9)	0.61
Cerebrovascular death (%)	279 (67.7)	386 (61.1)	**0.030**	165 (63.0)	199 (51.8)	**0.006**
HTN (%)	151 (37.0)	226 (36.2)	0.794	77 (29.5)	95 (24.8)	0.20
Serum creatinine (mg/dl)	0.9 ± 0.5	0.9 ± 0.41	0.82	0.90 ± 0.45	0.89 ± 0.36	0.93
**Post-transplantation**						
Cold ischemia time (hours)	18 ± 5.3	17.8 ± 5.6	0.58	17.8 ± 5.5	17.7 ± 5.6	0.58
Induction treatment (%)	257 (65.4)	434 (71.7)	**0.035**	145 (57.5)	238 (63.5)	0.16
Basiliximab (%)	88 (22.7)	148 (24.7)	0.49	55 (21.9)	90 (24.3)	0.56
Maintenance immunosuppression treatment (Tacrolimus) (%)	315 (77.2)	476 (77.3)	0.89	201 (76.7)	285 (74.4)	0.27
DGF (%)	116 (29.7)	180 (30.5)	0.83	67 (26.4)	104 (28.3)	0.65
AR (%)	55 (13.9)	74 (12.3)	0.50	40 (15.6)	47 (12.6)	0.29
**Graf failure, causes:** Chronic rejection Death Primary non-function Acute rejection Recurrence of ESRD Virus BK nephropathy Others	68 (15.6) 51 (11.7) 22 (5.1) 5 (1.1) 5 (1.1) 0 9 (2.1)	56 (8.4) 89 (13.4) 48 (7.2) 7 (1.1) 10 (1.5) 4 (0.6) 11 (1.7)	**0.014**	51 (18.4) 21 (7.6) 8 (2.9) 4 (1.4) 4 (1.4) 0 (0) 5 (1.8)	33 (8.1) 30 (7.4) 25 (6.1) 4 (1) 7 (1.7) 3 (0.7) 4 (1)	**0.003**
**Death causes:** Infection Cardiovascular Cancer Others	12 (2.7) 16 (3.6) 11 (2.5) 12 (2.8)	16 (2.4) 31 (4.6) 30 (4.5) 9 (1.3)	0.31	4 (1.4) 7 (2.4) 7 (2.4) 7 (2.4)	6 (1.4) 14 (3.2) 11 (2.5) 3 (0.7)	0.59

AR, acute rejection in the first 6 months after transplantation; BMI, body mass index; DBD, donation after brain death; DCD II, uncontrolled circulatory death donor (Maastricht II); DCD III, controlled circulatory death donor (Maastricht III); DGF, delayed graft function; DM, diabetes mellitus; ESRD, end-stage renal disease; HTN, high blood pressure; HLA, human leukocyte antigen; pre-Tx, pre-transplantation. Bold values denote statistical significance at the *p* < 0.05.

**TABLE 2:** Multivariate model to evaluate overall graft failure depending on recipients and donor variables in the whole study population (A) and in patients younger than 60-year series (B).

	Estimated coefficients	Standard error	HR (CI 95%)	*P*
**A**				
Recipient sex (male)	−0.137	0.184	0.872 (0.608–1.25)	0.46
Recipient age (year)	−0.003	0.009	0.997 (0.979–1.015)	0.75
Body mass index (%)	0.056	0.021	**1.058** (1.014–1.103)	**0.008**
HTN pre-transplant (yes)	0.046	0.279	1.047 (0.606–1.81)	0.87
HLA sensitization pre-transplant (yes)	0.996	0.281	**2.708** (1.56–4.7)	**<0.001**
Donor sex (male)	−0.098	0.177	0.907 (0.641–1.283)	0.58
Donor age (year)	0.020	0.007	**1.02** (1.005–1.034)	**0.007**
Brain death (yes)	0.147	0.205	1.158 (0.774–1.732)	0.48
Cold ischemia time (hours)	0.002	0.019	1.002 (0.964–1.041)	0.93
Acute rejection (yes)	0.462	0.208	**1.587** (1.056–2.383)	**0.026**
**B**				
Recipient sex (male)	−0.335	0.229	0.701 (0.447–1.099)	0.12
Recipient age (year)	−0.011	0.012	0.989 (0.965–1.013)	0.36
Body mass index (%)	0.033	0.026	1.034 (1.983–1.087)	0.20
HTN pre-transplant (yes)	0.338	0.359	1.403 (0.694–2.836)	0.35
HLA sensitization pre-transplant (yes)	0.916	0.306	**2.5** (1.347–4.55)	**0.002**
Donor sex (male)	−0.181	0.217	0.834 (0.545–1.276)	0.40
Donor age (year)	0.020	0.009	**1.021** (1.004–1.038)	**0.017**
Brain death (yes)	−0.026	0.250	0.974 (0.597–1.59)	0.92
Cold ischemia time (hours)	0.012	0.024	1.012 (0.965–1.061)	0.63
Acute rejection (yes)	0.458	0.248	1.58 (0.972–2.57)	0.06

Concordance: 0.65. HTN, high blood pressure; HLA, human leukocyte antigen. Concordance: 0.653. HTN, hypertension; HLA, human leukocyte antigen. Bold values denote statistical significance at the *p* < 0.05.

### Female recipients younger than 60 years of age

The distribution of patients depending on their age at the time of transplantation resulted as follows: 404 patients older than 60 years (37.1%) and 685 patients < 60 years (62.9%). Recipients < 60 years were 277 (40.4%) women and 408 (59.6%) men. In the bivariate analysis, no age differences were found between female and male recipients at the time of transplantation (Table 1). ESRD disease origin was different in female and male recipients, with similar results as in the whole study population. Smoking habits, as well as ischemic cardiomyopathy, were less prevalent in women than in men. HLA sensitization was more prevalent in women, without differences in the percentage of re-transplanted patients or the number of previous kidney transplants. Female recipients were transplanted more frequently with female, older donors or DBD. No differences were found depending on the type of donor, cold ischemia time, induction treatment, *de novo* or maintenance immunosuppressive treatment, DGF, or AR episodes. Graft function (measured by serum creatinine) was worse in female recipients than in male recipients only during the first 4 years of the follow-up (*p* < 0.05).

**FIGURE 2:**
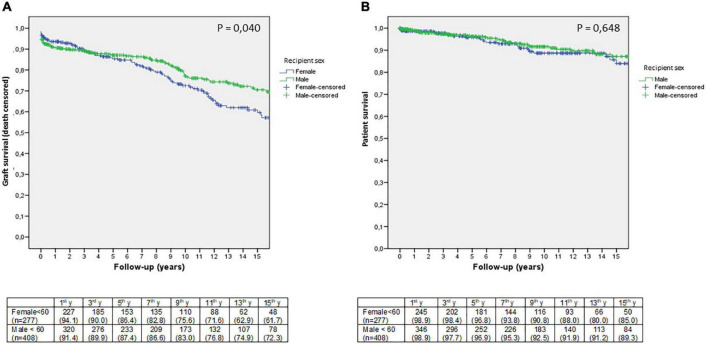
Kaplan-Meier estimates of death-censored graft **(A)** and patient survival **(B)** according to recipient sex in adults younger than 60 years. The number of patients at risk during follow-up is indicated in the table below the figures (*n* = 685).

Female recipients showed a decrease in graft survival (death censored) (log-rank, *p* = 0.037) later in the follow-up period, from the seventh year onward (Figure 2A). The incidence rate of graft failure was 4.1% in women (3.1% in men) per year. The main cause of graft failure was chronic rejection, which was more common in female recipients, while primary non-function was more common in male recipients (*p* = 0.003) (Table 1). Recipient sex was not an independent risk factor for graft failure in the Cox analysis. HLA sensitization before transplantation resulted in a 2.5-fold higher risk of graft failure than in non-sensitized patients (*p* = 0.027), followed by donor age (HR 1.021, *p* = 0.017) (Table 2). We analyzed the effect of pre-transplant sensitization status and donor age on graft survival, and the worst graft survival was found in pre-transplant sensitized patients who received a graft from a donor older than 60 years (*p* < 0.001). No differences in graft survival were found between sensitized patients transplanted with grafts from younger donors and non-sensitized recipients transplanted with grafts from donors older than 60 years (*p* = 0.845). Patient survival was similar in both men and women (log-rank, *p* = 0.850) (Figure 2B), as was the incidence rate of patient death (0.9%/year). The main cause of death was cardiovascular disease, followed by cancer, with no differences between female and male recipients.

## Discussion

In this study, we found that there are differences in graft survival between women and men in patients younger than 60 years, unlike in the older population. This fact led us to consider the need to develop different strategies to improve outcomes in this high-risk population.

Numerous differences related to the gender of the recipient have been described in the medical literature. First, the prevalence of CKD is higher in women than in men (7), a fact that may favor next-generation CKD (1, 2, 11). However, although the prevalence is higher in women at all stages of CKD, access to RRT is lower in female patients (2). These differences are related to several factors: the different etiology of ESRD in men and women, the protective effect of estrogens, better adherence to treatment and healthier lifestyle, and poverty or lack of health literacy in women (2, 12, 13). Finally, unequal access to transplantation for women and girls has also been described (4).

We designed this study to investigate whether post-transplant outcomes differ among men and women in our single-center cohort of renal transplant recipients. The ratio of female to male transplanted patients in our series was similar to that described in other studies, with almost 40% of female patients and 60% of male patients, supporting the differential need for RRT according to the gender of the recipient (4). When analyzing our entire study population, we found differences in demographic factors depending on the sex of the recipient, such as the cause of ESRD, with interstitial and polycystic kidney disease being most common in female recipients, while glomerular and vascular disease were most common in male recipients. In addition, male recipients were more likely to smoke and have a history of ischemic cardiomyopathy, both factors associated with a poorer prognosis. However, in the overall study population, we did not find any differences in graft or patient survival.

Although we started the study including all patients in our series, our main interest was to investigate the differences in the under-60 population who were more likely to need another transplant in case of graft failure. In this selected population, we did not find differences in patient survival depending on the sex of the recipient, as described by other authors, but we did find that women under 60 years of age had a higher risk of graft failure during long-term follow-up compared to men. In this subgroup of patients, the distribution of the origin of ESRD, ischemic cardiomyopathy, and smoking habits were similar to the overall study population. Women in the group of patients younger than 60 years had a higher prevalence of pre-transplant HLA sensitization than in the whole series. As the proportion of re-transplanted patients was the same, previous blood transfusions and pregnancies were the most likely reason for sensitization in female recipients (5, 14). Sensitization has been described as the main reason for women’s limited access to kidney transplantation (15, 16), and when women eventually receive a kidney transplant, it is also the main risk factor for graft failure, as we have shown in our study.

However, sensitization is not the only factor associated with poorer graft survival in women. Women received a higher proportion of grafts from female donors, older age, or DBD. Life expectancy is generally longer in women, and there is a high proportion of women among deceased donors in which cerebrovascular accidents are the most common cause of death (4). In addition, kidneys in women have a reduced nephron mass compared to those in men, which could be reduced even more in case of donors with extended criteria as a result of the aging process (17). On the contrary, the lower metabolic demand described for women, due to their smaller body size and weight, could mean a survival advantage for the graft over men in a donor with similar characteristics (4, 18–20). This approach may have been the main reason for the most frequent use of this type of donor in female recipients in our series. However, after analyzing these results, our experience shows that this theoretical survival advantage of women could reduce or even disappear in the case of donors with extended criteria, especially if the recipient has another strong risk factor for graft failure, such as HLA sensitization. Indeed, patients with pre-transplant HLA sensitization who received a graft from an old donor were those with a lower survival rate. So, in our experience, being a female recipient may have led to a negative bias in donor selection, negatively affecting prognosis, especially in younger women who have a higher percentage of HLA sensitization. Other factors such as DGF or AR were not associated with graft failure in recipients under 60 years of age. Induction treatment was used in a large proportion of patients in the entire series, not only in sensitized patients but also in patients at high DGF risk and low immunological risk with different doses. The high DGF risk might be the reason why induction was used more often in male recipients than in female recipients, but we would like to emphasize that the induction protocol was more intensive in sensitized patients than in patients in whom induction was used because of a high DGF risk.

This study has several limitations, mainly related to its monocentric and retrospective nature, although the data were collected prospectively by specially trained personnel. We presented data from our series, which included a large number of patients followed up over a long period of time, and this study allowed us to detect long-term differences between female and male recipients that might be underestimated by other studies or registries with a lower follow-up (6). However, the long follow-up time of the study is another factor that might increase the risk of bias, as differences only appear from the seventh year onward, so the results might be affected by many factors that we cannot even measure.

We do not have data on other gender differences described by other authors in female recipients, such as lower rates of pre-transplant medical screening, lower access to the waiting list, longer waiting list retention, or lower rates of kidney transplantation from deceased or living donors in women (4, 21–25). In our series, we found no differences in dialysis modality, although preemptive transplantation was less common in women.

The causes of differences related to recipient sex are multiple and complex, and we know less today about how to identify and resolve them. There are only a small number of studies, most of them monocentric, like ours, or focused on very specific issues (e.g., biological factors, personal finance, health literacy issues, and living donation) and rarely extended to other geographical locations or health systems (15, 20, 22–24, 26). In addition, it has already been described that health workers have limited ability to identify inequalities related to recipient sex, and these inequalities appear to be more difficult to identify than those related to financial issues, race or health literacy of kidney patients (22, 27, 28). In our study, we did not focus on financial issues because our public health system guarantees access to any treatment for the entire population, and difficulties in obtaining immunosuppressive treatment due to low income, as described for women in other countries or communities, are uncommon (14, 29, 30). We also ignore the impact of socio-cultural or financial problems on female patients’ access to our health system, as described in other countries with health systems similar to ours (28, 31, 32).

However, our study has allowed us to learn about the state of this issue in our center, where deceased donors are our main source of kidneys, with a high proportion of donors with expanded criteria, and these results could be representative of other transplant programs with similar characteristics and allocation policies to ours. The most important question for us is how to improve graft survival in adult female recipients younger than 60 years. In our opinion, we need protocols that include pre-transplant and post-transplant measures. Before transplantation, sensitized patients should be enrolled in specific programs to achieve the best HLA compatibility, such as the National Priority Allocation System for Hypersensitized Patients based on virtual crossmatching, which has shown excellent results in cadaveric donor transplantation in Spain and the development of similar programs at the regional level (33). Desensitization treatments could facilitate access of sensitized patients to living donor transplant programs through strategies such as ABO-incompatible transplantation or exchange of kidney pairs (5). After transplantation, the use of specific immunosuppressive treatment protocols for patients at higher immunological risk, taking biopsies to detect humoral rejection at earlier stages, increasing the number of medical checks to detect poor adherence in suspected cases, or using low-nephrotoxic immunosuppressive treatments with their further evaluation must be considered to reduce graft failure (34). Other interventions such as reducing the use of older donor grafts in these sensitized patients would be desirable, but the time on dialysis could be prolonged and patient survival could be compromised while waiting for a graft with a standard risk profile (35). We would like to emphasize that there are no differences in patient survival according to the sex of the recipient in long-term follow-up, in the whole study population and in the under-60 group, despite poorer graft survival in the latter. However, there is no doubt that we need to develop new strategies to improve long-term graft outcomes in the most vulnerable patient group, such as female recipients under 60 years of age because they have a higher proportion of pre-transplant HLA and are more likely to use expanded criteria donors compared to men. Part of the solution to this problem is to improve the living donor program at our center, as has been done in recent years. In addition, in recent months we have introduced a new computer system to optimize allocation based on an objective score, which will help us find the best recipient for each kidney transplant and avoid selection biases like this one based on the sex of the recipient.

In summary, no differences in graft and patient survival were found in the overall study population depending on the sex of the recipient, but the group of female recipients younger than 60 years had lower graft survival at longer follow-up than the male recipients. HLA sensitization and older donors were the main risk factors for poorer graft survival, with both factors being more pronounced in young female recipients. In female recipients younger than 60 years, strategies to improve outcomes are needed to avoid allocation bias and differences in graft survival depending on the gender of the recipient. These differences could be underestimated. Therefore, multicenter and high-quality studies are needed to improve our knowledge of this problem, find the best approach to avoid it, and, finally, improve our outcomes in long-term follow-up.

## Data availability statement

The raw data supporting the conclusions of this article will be made available by the authors, without undue reservation.

## Ethics statement

Ethical review and approval was not required for the study on human participants in accordance with the local legislation and institutional requirements. The patients/participants provided their written informed consent to participate in this study.

## Author contributions

AS, EG, and JK participated in the research design, the writing of the manuscript, the performance of the research, and in the data analysis. SB, CC, and VE participated in the research design and the writing of the manuscript. JP and PM analyzed the data. BV, MG, and EC made the figures and revised the manuscript. AA drafted and revised the manuscript. All authors approved the final version of the manuscript.

## Abbreviations

AR: acute rejection in the first 6 months after transplantation
BMI: body mass index
CI: confidence interval
CKD: chronic kidney disease
DBD: donation after brain death
DCD II: uncontrolled circulatory death donor (Maastricht II)
DCD III: controlled circulatory death donor (Maastricht III)
DGF: delayed graft function
DM: diabetes mellitus
ESRD: end-stage renal disease
HR: hazard ratio
HD: hemodialysis
HLA: human leukocyte antigen
HTN: high blood pressure
LD: living donor
PD: peritoneal dialysis
PRA: panel reactive antibody
PKD: polycystic kidney disease
< 60: adult younger than 60 years of age.
